# Pan-genome analysis and abiotic stress expression of the *SWEET* gene family in *Brassica napus*

**DOI:** 10.3389/fpls.2026.1846550

**Published:** 2026-05-26

**Authors:** Jinsong Xu, Jingdong Chen, Jinjia Long, Tianyuan Xue, Xuekun Zhang

**Affiliations:** 1 MARA Key Laboratory of Sustainable Crop Production in the Middle Reaches of the Yangtze River (Co-construction by Ministry and Province)/Hubei Key Laboratory of Waterlogging Disaster and Agricultural Use of Wetland, College of Agriculture, Yangtze University, Jingzhou, China; 2 Hubei Engineering Research Center for Protection and Utilization of Special Biological Resources in the Hanjiang River Basin, College of Life Science, Jianghan University, Wuhan, China

**Keywords:** abiotic stress, haplotype, pan-genome, *SWEET*, WGCNA

## Abstract

The *SWEET* (Sugars Will Eventually be Exported Transporters) gene family plays crucial roles in sugar transport, plant development, and abiotic stress responses. However, its pan-genomic characteristics and practical breeding potential in *Brassica napus* remain unclear. In this study, we systematically identified 96 *BnSWEET* genes across eight rapeseed genomes, revealing extensive presence/absence variations (PAVs) and clear classification into four subfamilies (Groups I–IV). Evolutionary analyses highlighted diverse selection pressures, with specific members (e.g., *BnSWEET37*) exhibiting strong signatures of positive selection (Ka/Ks > 1.0), while others were conserved under purifying selection. Haplotype analysis revealed that elite alleles of *BnSWEET5* are significantly associated with seed oil content, silique length, and germination vigor. Furthermore, Weighted Gene Co-expression Network Analysis (WGCNA) identified *BnSWEET47* as a core regulatory hub coordinating sugar partitioning and secondary metabolism. Transcriptomic profiling and RT-qPCR validation confirmed that *BnSWEET* genes, particularly those representing specific structural and evolutionary variants (e.g., the PAV-specific *BnSWEET26*), exhibit highly heterogeneous and tissue-specific temporal expression patterns under multiple abiotic stresses. Overall, this study elucidates the evolutionary and functional divergence of the *BnSWEET* family and proposes a practical breeding pathway—utilizing the identified elite haplotypes and key PAV variants for precision germplasm screening—to optimize source-sink balance and enhance abiotic stress resilience in *B. napus*.

## 1 Introduction

SWEET proteins are a class of facilitative transporters involved in sugar movement across cellular membranes. They were first identified as a novel transporter family by Chen et al (Chen et al., 2010). Subsequent work showed that *AtSWEET11* and *AtSWEET12* mediate sucrose efflux during apoplasmic phloem loading, establishing SWEET proteins as important components of long-distance assimilate transport (Chen et al., 2012). In contrast to proton-coupled SUT/SUC transporters, SWEETs generally mediate the transport of mono- or disaccharides along concentration gradients and are therefore important for intercellular carbon distribution (Chen, 2014; Eom et al., 2015). Structural studies further showed that eukaryotic SWEET proteins usually contain seven transmembrane helices, a feature closely related to their transport properties and evolutionary conservation (Tao et al., 2015). More recent studies have suggested that the functions of SWEET proteins extend beyond sugar transport itself and are closely connected with plant development and stress responses (Julius et al., 2017; Breia et al., 2021; Ji et al., 2022).

A growing number of studies have shown that *SWEET* genes participate in sugar allocation during plant development. In Arabidopsis, *AtSWEET11* and *AtSWEET12* are involved in sucrose export from leaves and phloem loading (Chen et al., 2012), whereas *AtSWEET11*, *AtSWEET12*, and *AtSWEET15* are successively expressed in the seed coat and endosperm to support embryo growth (Chen et al., 2015). In rice, *OsSWEET11* and *OsSWEET15* play important roles in grain filling and endosperm development (Yang et al., 2018). *SWEET9* has also been shown to be required for nectar secretion, indicating that members of this family are involved not only in assimilate transport but also in reproductive development (Lin et al., 2014). Taken together, these studies suggest that *SWEET* genes contribute to carbon partitioning across tissues and developmental stages and can ultimately affect plant growth and yield formation (Breia et al., 2021).

In addition to their developmental roles, *SWEET* genes are also associated with plant responses to biotic and abiotic stresses. Because SWEET proteins mediate sugar efflux, they can be exploited by pathogens as susceptibility factors for obtaining host carbon resources (Breia et al., 2021; Cheng et al., 2021). For example, TAL effector-induced expression of a *SWEET* gene increases bacterial disease susceptibility in cotton, whereas tonoplast-localized *AtSWEET2* restricts carbon loss from roots and limits Pythium infection (Chen et al., 2015; Cox et al., 2017). Increasing evidence also links *SWEET* family members to drought, salinity, cold, and heat responses. In Arabidopsis, loss of *AtSWEET11*/*12* affects freezing tolerance, and overexpression of *AtSWEET16* alters germination and stress tolerance. In rice, *OsSWEET13* and *OsSWEET15* are induced during ABA-related drought and salt responses (Klemens et al., 2013; Le Hir et al., 2015; Mathan et al., 2021). Genome-wide analyses in wheat and cotton further showed that the *SWEET* family has expanded substantially in polyploid crops and that many members display distinct stress-responsive expression patterns (Li et al., 2018; Qin et al., 2020). These observations point to a close connection between carbohydrate allocation and stress adaptation.

From an evolutionary perspective, the *SWEET* family is widely distributed in plants and has undergone repeated duplication and functional divergence. Comparative studies have shown that angiosperm *SWEET* genes can be grouped into several conserved clades, but members within these clades often differ in expression pattern and biological role (Li et al., 2018). Although the basic structural features of SWEET proteins are highly conserved and support their common transport function, differences in duplication history, sequence divergence, and cis-regulatory elements likely contributed to the specialization of individual members (Tao et al., 2015; Ji et al., 2022). In *B. napus*, Jian et al. (2016) identified 68 *SWEET* genes and analyzed their phylogenetic relationships, chromosomal distribution, gene structures, conserved motifs, cis-elements, and expression profiles, providing the first genome-wide description of this family in rapeseed. However, that study was based on a single reference genome and could not fully reflect variation in *SWEET* family composition among different rapeseed accessions.

As a young allotetraploid species derived from the hybridization between *B. rapa* and B. oleracea, *B. napus* has undergone polyploidization, gene loss and retention, homoeologous exchange, and extensive structural variation during its evolution. Consequently, gene copy number, presence/absence status, and expression profiles can vary significantly among different genotypes (Song et al., 2020). Recent pan-genomic studies have established a robust framework for examining this diversity. The *B. napus* pan-genome has revealed widespread presence/absence variation (PAV) and numerous large-effect genomic changes; subsequent research has demonstrated that these structural variants influence gene expression and agronomic traits at the population level (Song et al., 2020; Zhang et al., 2024b) Pan-genomic approaches have also been successfully applied to specific rapeseed gene families, such as the R2R3-MYB family, uncovering critical differences among core, variable, and accession-specific gene clusters (Fan et al., 2025).

In contrast, a comprehensive pan-genomic analysis of the SWEET family in *B. napus* is currently lacking. Previous bioinformatic studies on rapeseed SWEET genes have primarily relied on a single reference genome, providing only a “static snapshot” that fails to capture the extensive single nucleotide polymorphisms (SNPs) and presence/absence variations (PAVs) across diverse cultivars. This “genomic blind spot” hinders a thorough understanding of how the SWEET family has undergone functional diversification and differential retention following allopolyploidization—scientific questions that cannot be fully addressed from a single-genome perspective. In particular, the conservation, homoeologous gene cluster composition, and copy number variations of this family remain poorly understood. To address these gaps, we performed a pan-genomic analysis of the SWEET family across eight *B. napus* genomes. By surmounting the limitations of a single reference genome, we successfully identified 37 previously unannotated BnSWEET members and characterized their genomic distribution. By integrating abiotic stress-related expression data, we systematically analyzed the family composition, phylogenetic relationships, and responsive patterns. This study establishes a foundation for understanding the conservation and diversification of SWEET genes in rapeseed and provides promising candidate genes for enhancing stress tolerance and optimizing assimilate partitioning in future breeding programs.

## 2 Results

### 2.1 Pan-genome-wide BnSWEET identification

**Figure 1:**
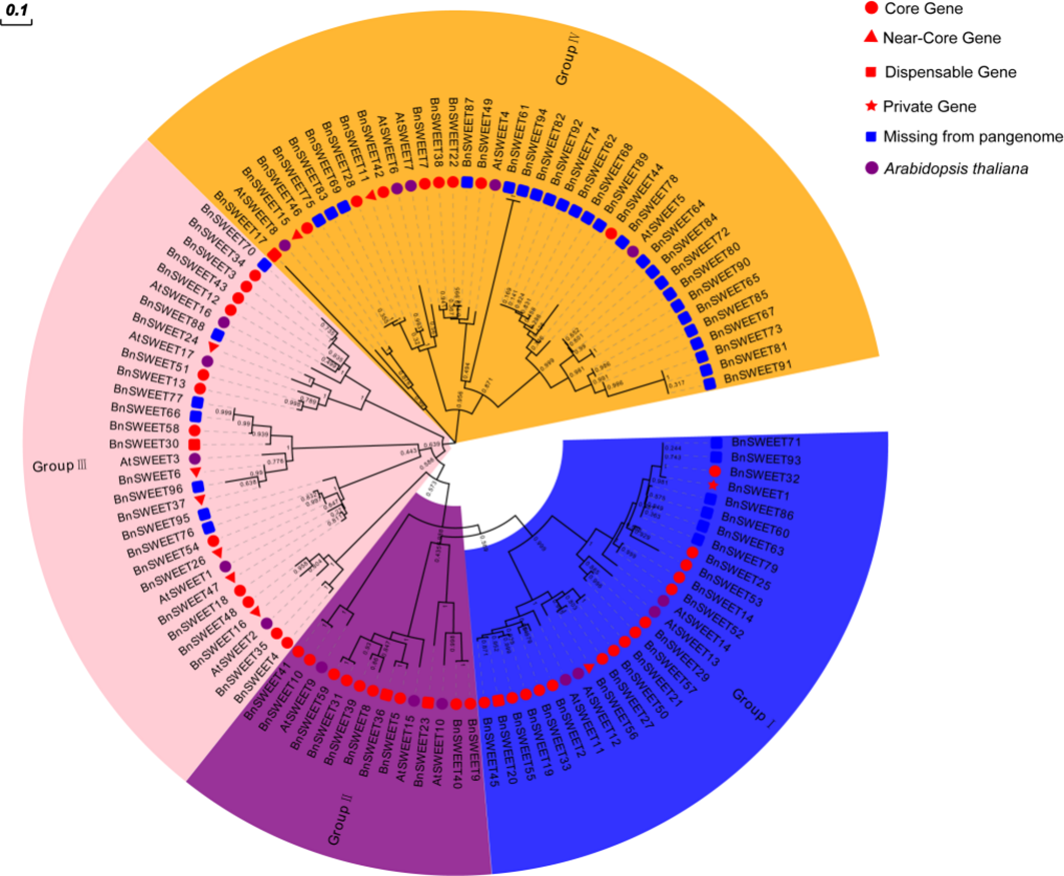
Phylogenetic tree of *B. napus* and Arabidopsis *SWEET* genes.

**Figure 2:**
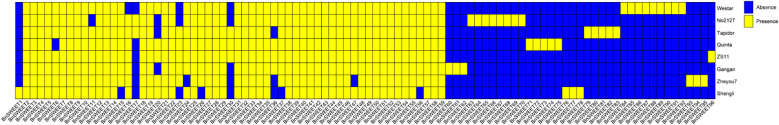
Heatmap showing the presence/absence patterns of 96 *SWEET* genes across 8 *B. napus* varieties. Genes within blue boxes represent absence variations, while those within yellow boxes indicate presence.

This study identified a total of 96 *BnSWEET* genes across the *B. napus* (rapeseed) pan-genome. These comprised 44 core genes (present in all accessions), 5 dispensable genes, 9 near-core genes, 1 private gene (BnSWEET1, assigned to Group I subfamily), and 37 unannotated genes specific to the pan-genome (i.e., absent from the reference assembly) (**Figure 1**; **Supplementary Table 1**; **Supplementary Figure 1**). Phylogenetic analysis was performed using BnSWEET protein sequences derived from Song et al (Song et al., 2021) pan-genome annotations. Following the established classification of Arabidopsis SWEET proteins, the *BnSWEET* genes were grouped into four subfamilies: Group I (24 genes), Group II (11 genes), Group III (26 genes), and Group IV (35 genes). Presence/absence variation (PAV) analysis across eight representative *B. napus* accessions, visualized in the accompanying heatmap, revealed pronounced accession-specific patterns (**Figure 2**). While the majority of *BnSWEET* genes were conserved across most lines, certain members exhibited clear cultivar-specific presence. For instance, *BnSWEET17* was detected exclusively in No2127 and Tapidor, whereas *BnSWEET30* was unique to Tapidor, Quinta, and ZS11 (absent in the remaining accessions).

Quantification of the dispensable (variable) genes showed substantial differences in their distribution among the accessions: Quinta and ZS11 each harbored 4, Tapidor had 3, while No2127, Westar, Gangan, and Zheyou7 each contained 2, and Shengli possessed only 1.These observed patterns of differential *BnSWEET* gene distribution, particularly among the dispensable fraction of the pan-genome, strongly suggest a potential association with accession-specific physiological traits, environmental adaptation, yield components, or other agronomically important phenotypes in diverse rapeseed varieties.

The phylogenetic tree was constructed using the Neighbor-Joining (NJ) method with 1000 bootstrap replicates. Bootstrap values are indicated at the key nodes.

### 2.2 BnSWEET Is subjected to different selection pressures among B. napus varieties

**Figure 3:**
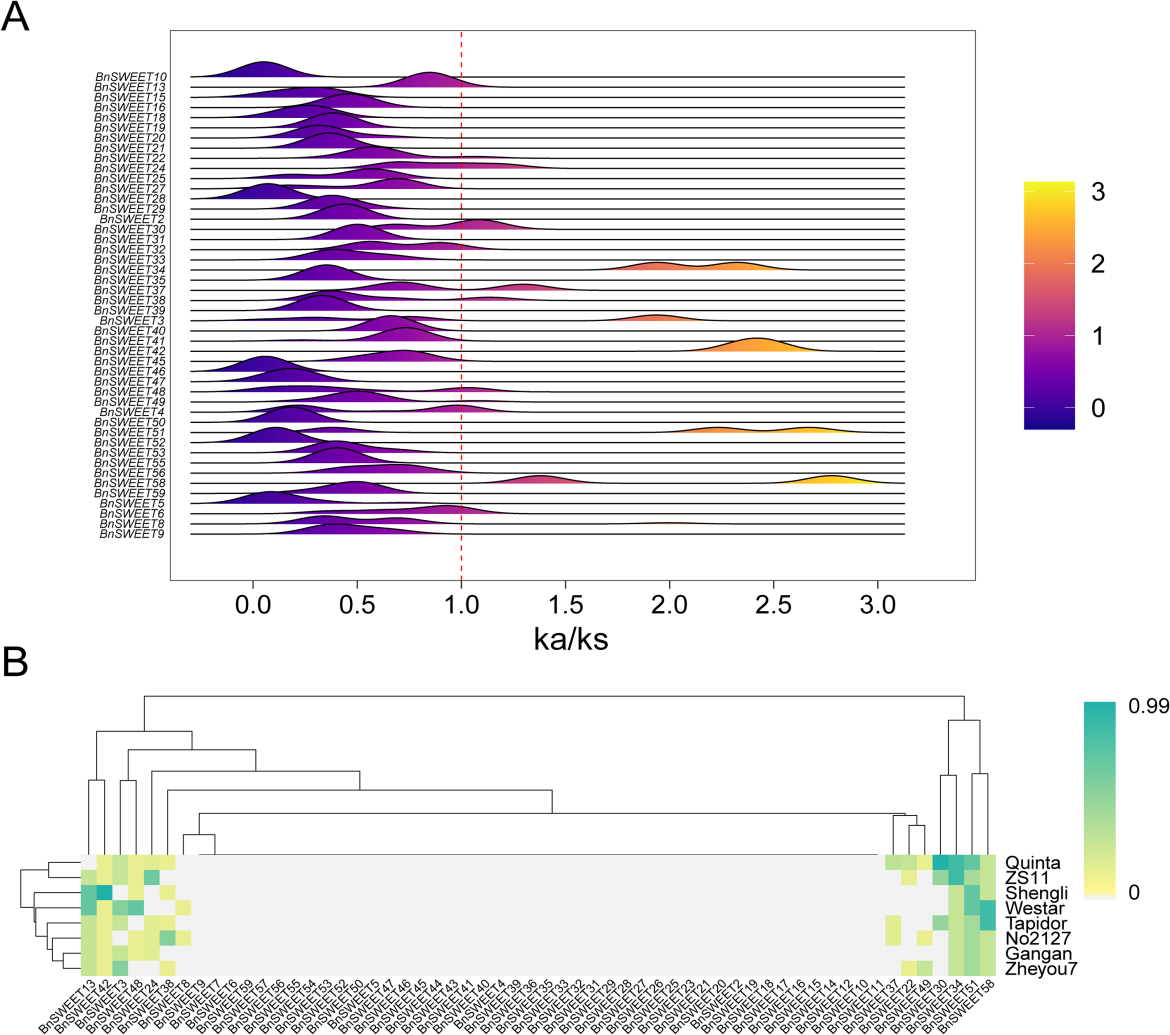
Ka/Ks values of BnSWEET. **(A)** Distribution of Ka/Ks values of BnSWEET in 8 *B. napus* varieties. The x-axis represents the range of the Ka/Ks values, while the height and color of the peaks in the figure indicate the number of gene accumulations in different *B. napus* varieties. **(B)** Heatmap of the frequency of occurrence of different *B. napus* varieties for each SWEET with a Ka/Ks ratio > 1. The x-axis represents *B. napus SWEET* genes, and the y-axis represents different *B. napus* varieties. The colored blocks in the figure indicate the ratio of *SWEET* genes with a Ka/Ks value greater than 1 in different varieties.

To investigate the selective pressures acting on the *BnSWEET* gene family during its evolution, nonsynonymous-to-synonymous substitution rate ratios (Ka/Ks) were calculated across eight *B. napus* genomes, yielding values ranging from 0 to 2.77716 (**Figure 3A**). Our analysis revealed highly diversified selection patterns across the four phylogenetic groups: Group I and the majority of Group II (e.g., *BnSWEET5* and *BnSWEET9*) exhibited Ka/Ks ratios significantly below 1.0, reflecting intense purifying selection and strong functional conservation. In contrast, Groups III and IV emerged as “hotspots” for adaptive evolution; specifically, members such as *BnSWEET30*, *BnSWEET51*, and *BnSWEET58* in Group III, and *BnSWEET42* in Group IV, consistently displayed Ka/Ks > 1 (with *BnSWEET58* reaching a maximum of 2.77716), indicating robust signatures of positive selection. While certain genes like *BnSWEET6* and *BnSWEET7* maintained conserved functions under purifying selection, the prominent positive selection signals in Groups III and IV underscore the functional diversification and adaptive potential of the BnSWEET family in response to the complex environmental challenges faced by allotetraploid rapeseed. Furthermore, the distribution and frequency of positive selection across the eight *B. napus* varieties were visualized in a heatmap (**Figure 3B**). The analysis revealed that positive selection is not uniformly distributed across the gene family or the accessions. Certain genes, such as *BnSWEET58*, *BnSWEET42*, and *BnSWEET34*, exhibited a high frequency of Ka/Ks > 1 across almost all tested varieties, suggesting they have been subject to consistent and strong adaptive pressure during the expansion of *B. napus*. In contrast, other members like *BnSWEET8*, *BnSWEET22*, and *BnSWEET30* showed variety-specific positive selection patterns, appearing in only a subset of accessions. This variation in the frequency of Ka/Ks > 1 suggests a nuanced evolutionary landscape where specific *SWEET* genes may contribute to the localized adaptation of different rapeseed varieties to diverse environments or breeding selection.

### 2.3 Collinearity analysis of the BnSWEET gene family across eight rapeseed cultivars

**Figure 4:**
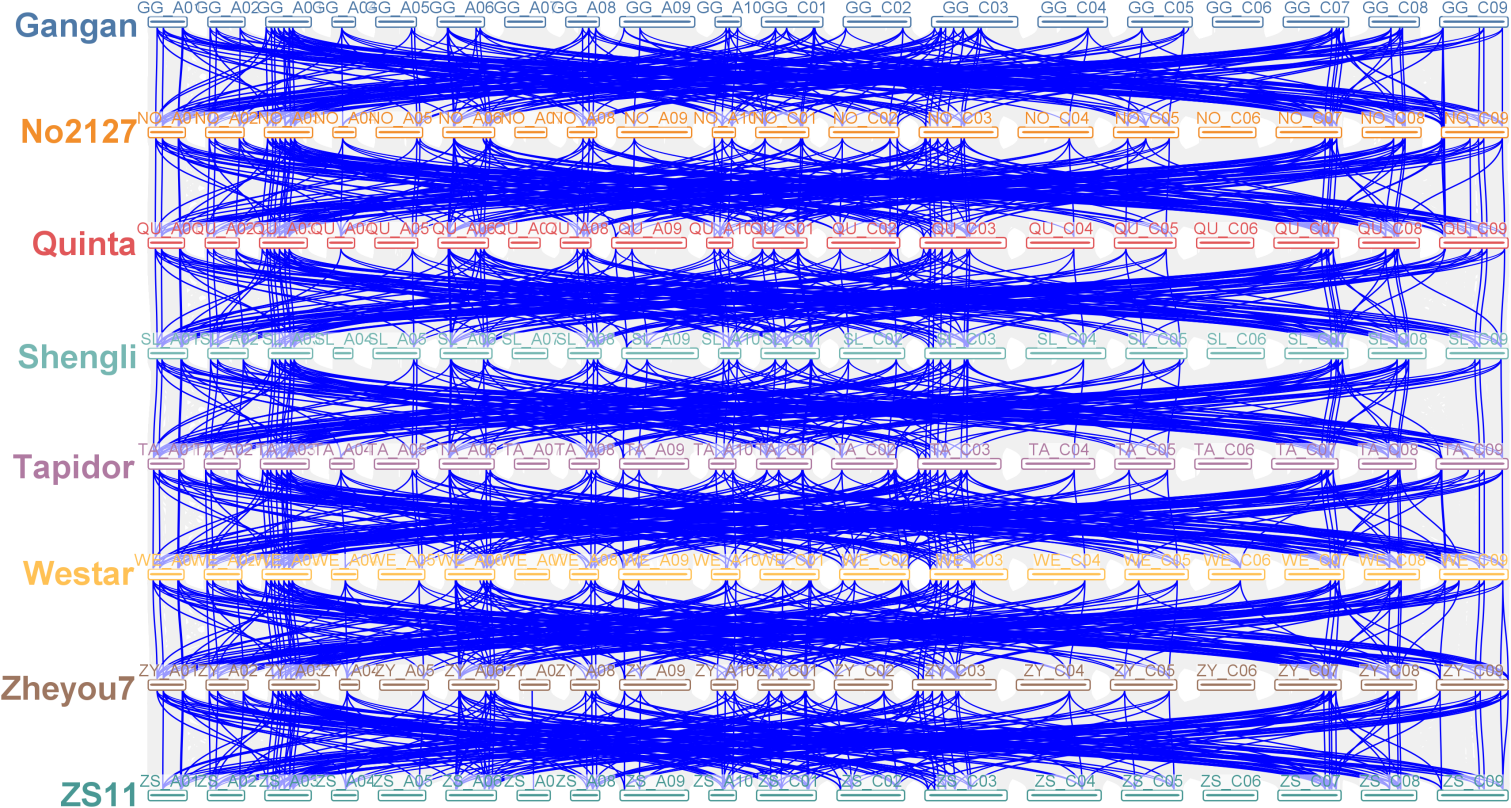
Collinearity analysis of *BnSWEET* genes in eight rapeseed.

We conducted a collinearity comparison of the *BnSWEET* genes across eight rapeseed cultivars (**Figure 4**). The results showed that 537 homologous gene pairs were identified between Gangan and No2127; 567 pairs between Quinta and No21217; 502 pairs between Shengli and Quinta; 480 pairs between Tapidor and Shengli; 541 pairs between Westar and Tapidor; and 528 pairs between Zheyou7 and Westar. In addition, 531 homologous gene pairs were detected between ZS11 and Zheyou7 (**Supplementary Table 2**). These findings indicate that a large number of homologous correspondences are shared among different cultivars, reflecting strong conservation and structural stability of the genome during evolution.

### 2.4 Comparative analysis of evolutionary characteristics of the BnSWEET family across different rapeseed cultivars

**Figure 5:**
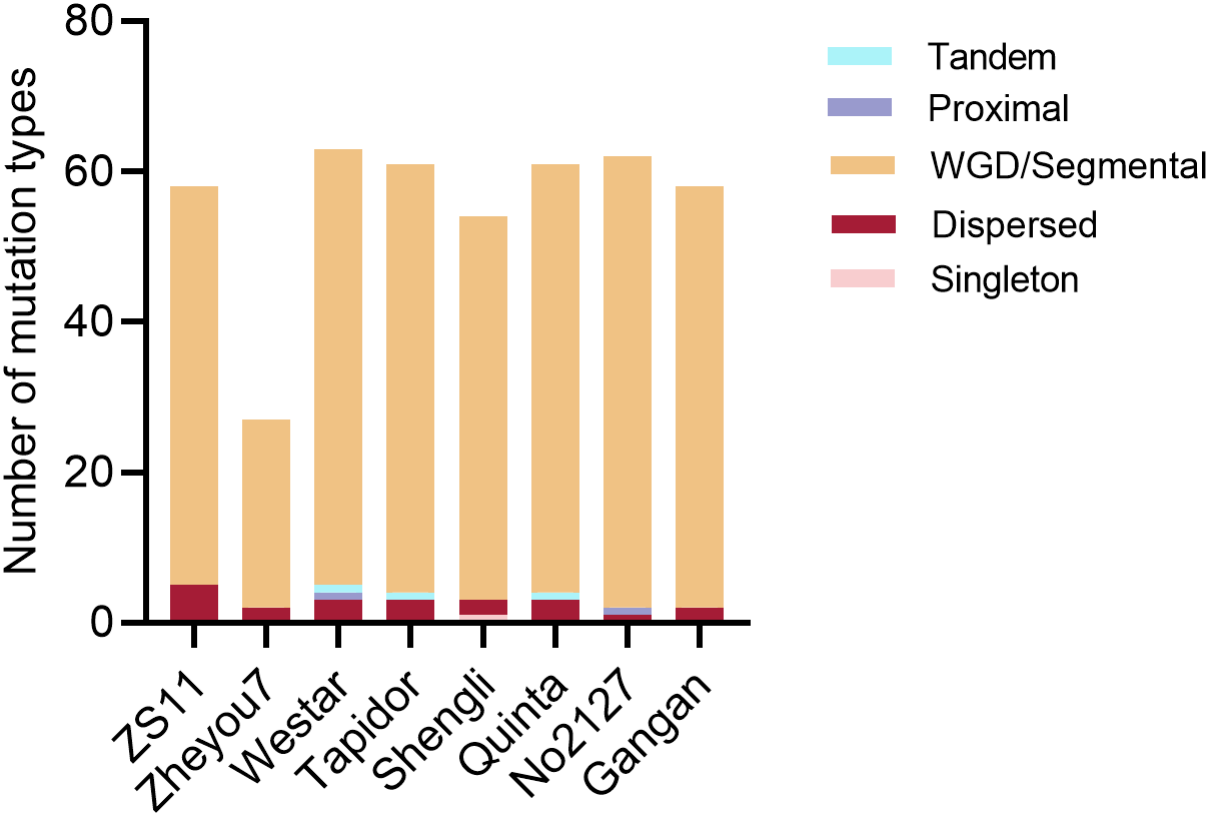
Duplication modes of the *BnSWEET* gene family across eight *B. napus* cultivars.

Comparative genomic analysis across eight *B. napus* cultivars identified whole-genome duplication (WGD) or segmental duplication as the primary drivers of *BnSWEET* family expansion. While the total gene count remained conserved (54–63 members, excluding Zheyou7), WGD-derived genes consistently predominated, accounting for 89.5% to 96.8% of the family across all genomes (**Figure 5**; **Supplementary Table 3**). Despite this stability, subtle evolutionary divergences were observed: Westar exhibited the most diversified pattern, uniquely harboring all four duplication types (WGD, dispersed, proximal, and tandem), suggesting more active localized duplication mechanisms post-polyploidization. In contrast, ZS11 and Gangan showed evolutionary stasis, lacking both tandem and proximal events, while Shengli was the sole accession to retain a singleton gene. Although Zheyou7 yielded fewer genes (27)—likely due to assembly or annotation constraints—its WGD-derived core remained stable at 92.6%. Overall, the “stable core with sporadic local variations” indicates that *BnSWEET* expansion primarily stems from shared allopolyploidization history rather than independent tandem bursts or cultivar-specific pressures.

### 2.5 Impact of BnSWEET gene variations on seed oil content, silique length and germination rate

**Figure 6:**
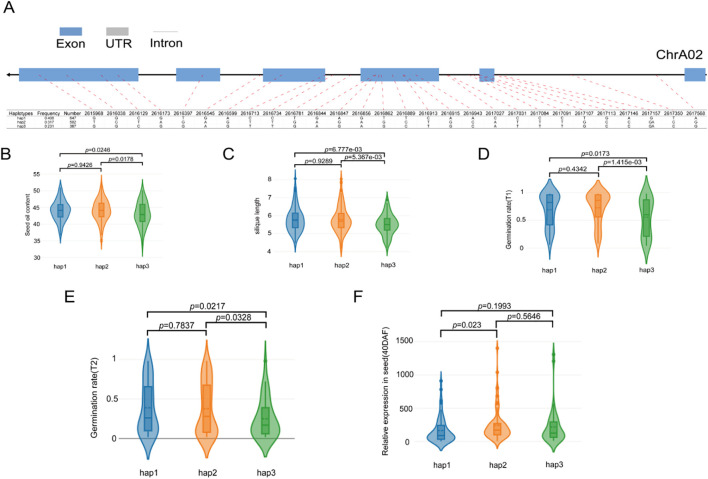
Effects of *BnSWEET5* haplotypes on seed oil content (SOC), silique length (SL) and germination rate major haplotypes of BnSWEET5: hap1, hap2 and hap3. **(B)** Comparison of seed oil content (SOC) among accessions carrying different *BnSWEET5* haplotypes. **(C)** Comparison of silique length (SL) among accessions carrying different *BnSWEET5* haplotypes **(D, E)** Comparison of thousand Germination rate(T1-T2) among accessions carrying different *BnSWEET5* haplotypes. **(F)** Relative expression analysis of *BnSWEET5* in seeds at 40 days (40 DAF).

To further explore the role of *BnSWEET* genes in regulating seed oil content, silique length, and germination rate across different rapeseed varieties, we performed sequence variation and haplotype analyses. In 1,626 rapeseed accessions, 28 sequence variants were identified in the *BnSWEET5* gene, including 27 SNPs and 1 InDel, which grouped into three major haplotypes (hap1, hap2, and hap3) (**Figure 6A**). Comparative analysis revealed that accessions carrying hap3 had significantly lower seed oil content than those with hap1 or hap2 (**Figure 6B**). Similarly, hap3 accessions showed reduced silique length (**Figure 6C**) and lower germination rates (**Figures 6D, E**) compared with hap1 and hap2. Additionally, accessions carrying hap2 exhibited significantly higher *BnSWEET5* expression at 40 days of seed development than those carrying hap1 (**Figure 6F**). Based on a large-scale diversity panel of 1,626 *B. napus* accessions, the linkage disequilibrium (LD) heatmap (**Supplementary Figure 2**) revealed a robust and conserved LD block (approximately 1.8 kb; A02: 2,615,915–2,617,686 bp) with R2 > 0.8 that almost completely encompasses the gene body of BnSWEET5. Notably, LD values decayed rapidly (R^2^ < 0.2) immediately beyond the gene boundaries. LD heatmap analysis indicates that the majority of sequence variants within BnSWEET5 are situated within this strong LD block; such high linkage consistency suggests that these intragenic variations exhibit a strong tendency toward co-segregation during population inheritance, thereby forming stable haplotype structures.

**Figure 7:**
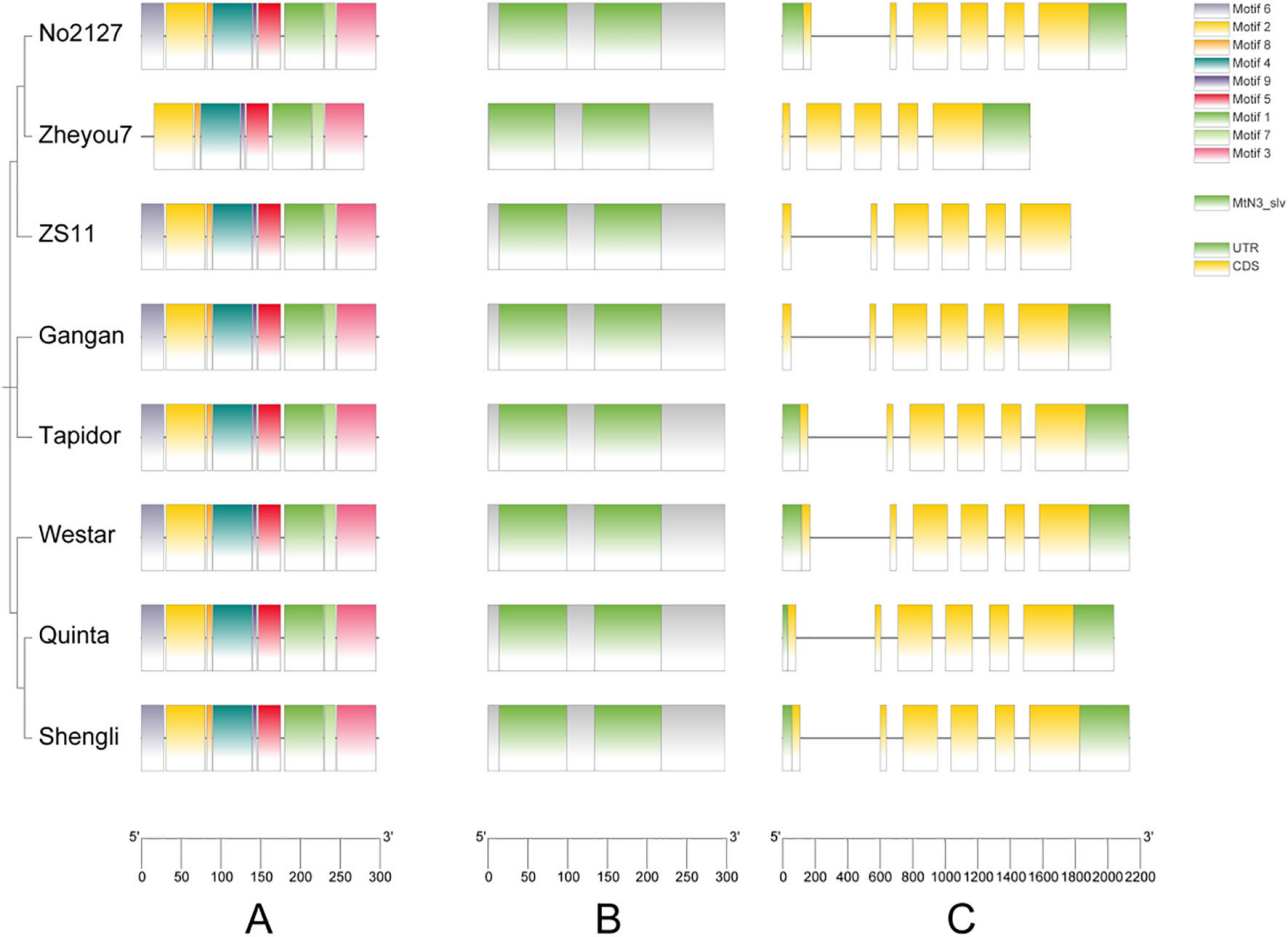
Comparative analysis of gene structure, motifs, and domains of *BnSWEET5* across eight *B. napus* genomes. **(A)** Gene structure. By using MEME Suite to search for motifs in the *BnSWEET5* gene, nine motifs were found to be conserved across different *B. napus* varieties. **(B)** Conserved domains: Demonstrates the stability of the MtN3 (MtN3/saliva) domain, which is a core characteristic of the *SWEET* family. **(C)** The structure of *BnSWEET5* across 8 different *B. napus* varieties. The structural differences in the gene occur in the 3′ untranslated region. *BnSWEET5* has an additional 3′ untranslated region in the *B. napus* varieties Zheyou7.

To investigate whether SNP variations affect the gene structure of *BnSWEET5*, we examined its architecture across eight *B. napus* genomes (**Figure 7**). The results showed that in No2127, Tapidor, Westar, Quinta, and Shengli, the domain composition, exon number, and 5′ untranslated region (UTR) of *BnSWEET5* were conserved. In contrast, ZS11 displayed variation in the 5’ UTR, which contained two fewer repeat units than the other five cultivars. In Zheyou7, more pronounced differences were observed: the domain composition, exon number, and both 5’ and 3’ UTRs differed, including the loss of motif 6, and each UTR as well as the exon region contained one fewer repeat unit compared with No2127, Tapidor, Westar, Quinta, and Shengli.

### 2.6 Transcriptome expression pattern analysis of BnSWEET

**Figure 8:**
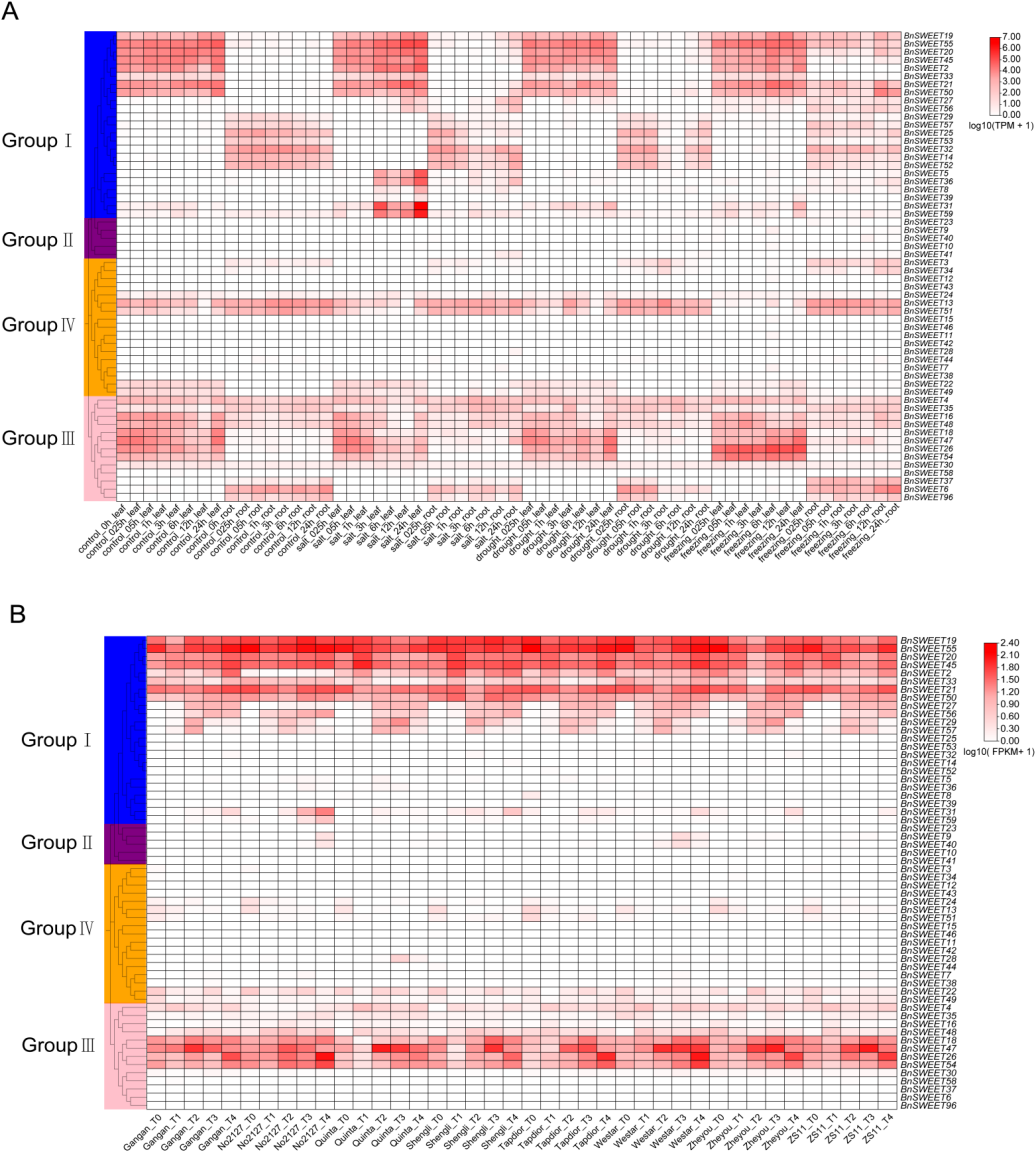
Expression patterns of *BnSWEET* genes. **(A)** Expression profiles of *BnSWEET* genes under various abiotic stress treatments. **(B)** Spatiotemporal expression of *BnSWEET* genes in eight *B. napus* accessions across different developmental stages.

To investigate the expression patterns of *BnSWEET* genes under abiotic stress conditions, we analyzed the expression profiles of the *SWEET* gene family in *B. napus* based on the ZS11 reference genome across different abiotic stress treatments, time points, and tissues (leaves and roots). The abiotic stresses examined included control (CK), salt stress (200 mmol·L^-1^ NaCl), drought stress (natural air-drying), freezing stress (-4 °C treatment followed by recovery at 25 °C), and osmotic stress (300 mmol·L^-1^ mannitol). The results were visualized in a heatmap (**Figure 8A**).

The analysis revealed that *SWEET* genes can be classified into four subfamilies: Group I, Group II, Group III, and Group IV, with each subfamily exhibiting distinct expression patterns across different stress treatments and time points. Group I contained the largest number of gene members. Under control conditions, *BnSWEET19*, *BnSWEET55*, *BnSWEET20*, *BnSWEET45*, *BnSWEET2*, *BnSWEET50*, and *BnSWEET21* showed relatively high expression levels in leaves, and their expression remained elevated following salt, drought, and osmotic stress treatments. In roots, *BnSWEET29*, *BnSWEET57*, *BnSWEET25*, *BnSWEET53*, *BnSWEET32*, *BnSWEET14*, and *BnSWEET52* displayed high expression in the control group and maintained elevated levels after the three stress treatments. Additionally, *BnSWEET5*, *BnSWEET36*, *BnSWEET31*, and *BnSWEET59* exhibited significantly high expression in roots specifically under salt stress. Members of Group II (e.g., *BnSWEET23* and *BnSWEET9*) consistently showed low expression levels under both control and all abiotic stress conditions. The overall expression pattern of Group III was similar to that of Group I: *BnSWEET18*, *BnSWEET47*, *BnSWEET26*, and *BnSWEET54* displayed relatively high expression in leaves under control conditions and maintained high levels after the three abiotic stress treatments, while *BnSWEET6* and *BnSWEET96* showed high expression in roots under control conditions and remained elevated post-stress. Notably, *BnSWEET30* exhibited high expression in roots at various time points across the three abiotic stresses and displayed clear upregulation in leaves at certain time points, suggesting its potentially critical role in the abiotic stress response of *B. napus*. In contrast, members of Group IV generally exhibited low overall expression levels, with only *BnSWEET13* and *BnSWEET51* showing relatively high expression in roots under control conditions, which was maintained at various time points across the three abiotic stress treatments. These findings indicate that the *SWEET* gene family in *B. napus* displays tissue-specific and diverse stress-responsive expression patterns under abiotic stresses, with clear functional divergence among different subfamilies and individual members.

Using the ZS11 reference genome of *B. napus*, the expression patterns of the *SWEET* gene family were analyzed across different abiotic stress treatment time points and tissues (leaves and roots), with results visualized in a heatmap (**Figure 8B**). The analysis revealed significant expression differences among *SWEET* genes at various abiotic stress treatment time points. In Group I, B*nSWEET27*, *BnSWEET56*, *BnSWEET29*, and *BnSWEET57* exhibited high expression in certain genotypes at specific sowing time points; *BnSWEET19*, *BnSWEET55*, *BnSWEET20*, *BnSWEET45*, *BnSWEET21*, and *BnSWEET50* maintained consistently high expression across all stages in all eight genotypes, while *BnSWEET2* and *BnSWEET33* showed elevated expression in the majority of stages. In contrast, most members of Group II and Group IV displayed low expression levels under both control and abiotic stress conditions, with only a few genes—such as *BnSWEET9* and *BnSWEET40* in Group II, and *BnSWEET24* and *BnSWEET13* in Group IV—showing upregulation at specific sowing stages in certain genotypes. Group III generally exhibited lower overall expression levels, although *BnSWEET18*, *BnSWEET47*, *BnSWEET26*, and *BnSWEET54* maintained consistently high expression across all stages in all eight genotypes.

### 2.7 WGCNA-based identification of stage-specific co-expression networks and hub genes

**Figure 9:**
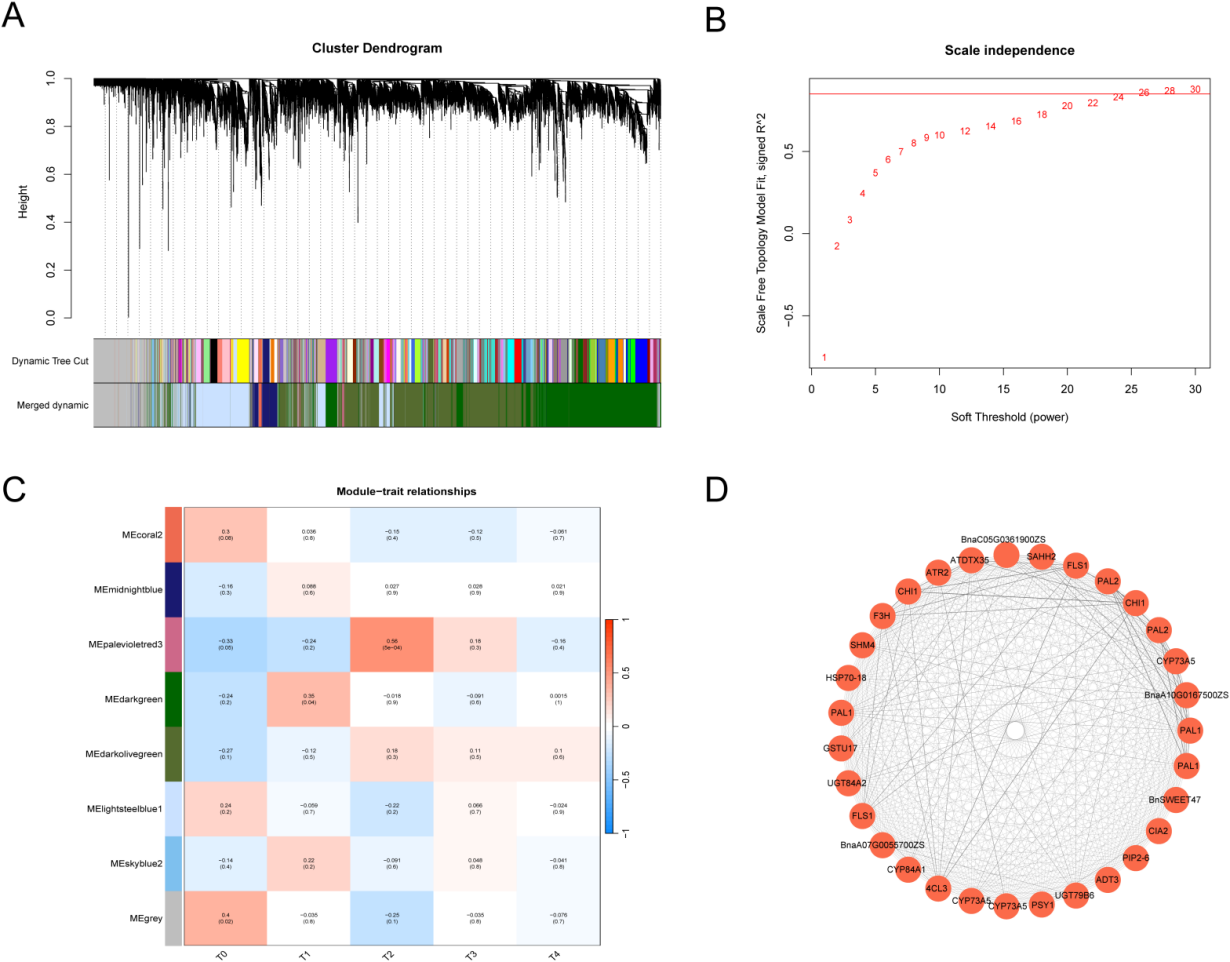
WGCNA-based identification of co-expression networks and hub genes across developmental stages in *B. napus*. **(A)** Hierarchical clustering dendrogram of genes and assignment of co-expression modules. **(B)** Determination of the optimal soft-thresholding power (β=26) to ensure a scale-free network topology. **(C)** Heatmap of module-trait relationships across five developmental stages (T0–T4). Numbers within the cells represent Pearson correlation coefficients and the corresponding p-values (in parentheses). **(D)** Visualization of the core co-expression network within the palevioletred3 module, highlighting the top 30 hub genes based on intramodular connectivity alongside *BnSWEET47*.

To explore the potential regulatory networks across five developmental stages (T0–T4) in eight Brassica napus cultivars, a Weighted Gene Co-expression Network Analysis (WGCNA) using a soft threshold power of 26 successfully identified eight distinct gene co-expression modules (**Figures 9A, B**). Module-trait relationship analysis revealed that these gene modules exhibited significant stage-specific associations. Notably, the palevioletred3 module showed a highly significant positive correlation with the T2 stage (r = 0.56, p = 5×10^-4^), and the darkgreen module was significantly positively correlated with the T1 stage. Meanwhile, the coral2 and grey modules were primarily positively associated with the initial T0 stage (**Figure 9C**).Further visualization of the core interaction network within the palevioletred3 module (comprising the top 30 genes by connectivity alongside *BnSWEET47*) revealed that the sugar transporter gene *BnSWEET47* shared an extremely tight co-expression relationship with multiple key enzyme genes involved in flavonoid biosynthesis, including PAL1/2, CHI1, F3H, FLS1, and CYP73A5 (**Figure 9D**). This finding strongly implies the existence of a crucial synergistic regulatory mechanism between BnSWEET-mediated carbohydrate partitioning and the synthesis and accumulation of secondary metabolites (e.g., flavonoids) during specific developmental stages in *B. napus*.

### 2.8 Temporal expression analysis of six representative BnSWEET genes under salt, alkali, and drought stresses

**Figure 10:**
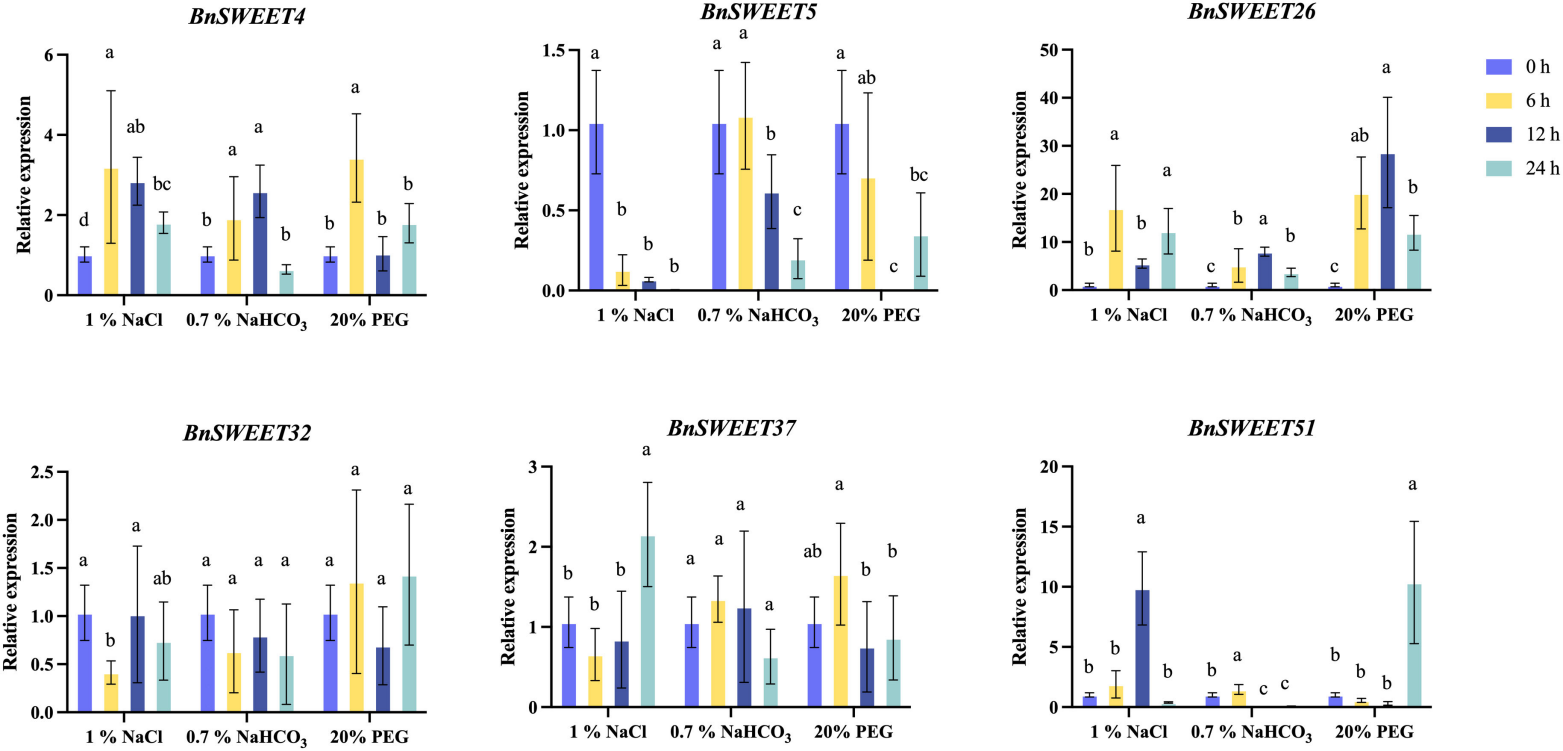
The relative expression levels of six representative *BnSWEET* genes in *Brassica napus* leaves were detected by RT-qPCR under different abiotic stress treatments: salt stress (1.0% NaCl), alkali stress (0.7% NaHCO_3_), and drought stress (20% PEG). Data represent the mean ± standard error (SE) of three biological replicates. Different lowercase letters indicate significant differences among treatment time points for the same gene (LSD test,P <0.05).

To validate the genomic and functional diversity of the *BnSWEET* family, six representative genes were selected for RT-qPCR analysis based on their trait associations, structural variations, and evolutionary signatures (**Figure 10**). Specifically, *BnSWEET5* was identified as a key haplotype gene associated with seed quality, while *BnSWEET26* represents a typical presence/absence variation (PAV) locus. Additionally, *BnSWEET37* was included as a candidate undergoing positive selection (Ka/Ks > 1.0). By incorporating members from Group I (*BnSWEET32*), Group III (*BnSWEET4*), and Group IV (*BnSWEET51*), this selection ensured broad phylogenetic coverage across the family.

To investigate the dynamic response characteristics of the *BnSWEET* family under abiotic stresses, six representative members were selected, and their temporal expression patterns in leaves under salt (1.0% NaCl), alkali (0.7% NaHCO_3_), and drought (20% PEG) stresses were detected via RT-qPCR. The results revealed significant differential responses among the genes to various stresses. Under salt stress, *BnSWEET4* and *BnSWEET51* peaked at 6 h and 12 h, respectively, followed by significant downregulation; *BnSWEET5* exhibited a continuous decrease; *BnSWEET37* was upregulated with a delay until 24 h; whereas *BnSWEET26* and *BnSWEET32* displayed opposite expression trends—specifically, *BnSWEET26* expression increased significantly at 6 h and decreased at 12 h before stabilizing, while *BnSWEET32* decreased significantly at 6 h and increased at 12 h before stabilizing. Under alkali stress, *BnSWEET4* and *BnSWEET51* were upregulated at 6 h and then declined; *BnSWEET26* and *BnSWEET5* were downregulated in the middle to late stages; and *BnSWEET32* and *BnSWEET37* showed no significant changes. Under drought stress, *BnSWEET4* and *BnSWEET37* peaked rapidly and then recovered; *BnSWEET26* gradually decreased after upregulation at 6 h; and *BnSWEET51* showed a delayed significant increase at 24 h. These results indicate that *BnSWEET* family members exhibit highly heterogeneous temporal expression patterns in response to salt, alkali, and drought stresses.

## 3 Discussion

SWEET proteins play important roles in sugar efflux, source-sink allocation, seed filling, and stress adaptation in plants. Previous studies have shown that this family is widely involved in sugar transport-related processes in many species. However, *SWEET* families are not identical across crops, and clear differences can occur in member number, evolutionary divergence, and expression pattern. For example, 23 *HvSWEET* genes were identified in barley and classified into four clades, and some members showed expression divergence and signatures of selection during domestication and improvement. Studies in Brassica juncea and cucumber also found that, although the overall family structure of *SWEET* genes is relatively conserved, different members can vary substantially in tissue expression and stress response (Yue et al., 2023; Heng et al., 2024). This suggests that the *SWEET* family is conserved at the overall structural level, whereas the specific members contributing to particular functions may differ among species.

In this study, a total of 96 *BnSWEET* genes were identified from the *B. napus* pan-genome. This number is higher than the number of family members typically recovered from a single reference genome, suggesting that pan-genome analysis offers clear advantages for capturing accession-specific or previously unannotated members. Phylogenetic analysis showed that these *BnSWEET* genes could be assigned to the same four major clades reported in other plants, indicating that the basic evolutionary framework of the family is largely conserved in rapeseed. At the same time, the PAV analysis revealed that certain members were not evenly distributed among accessions. This suggests that the *BnSWEET* family consists of both relatively stable core members and more variable (dispensable) members. Similar patterns have been observed in barley and *B. juncea*, where specific *SWEET* genes differ in genomic organization, selective pressure, or stress-related expression (Yue et al., 2023). While these variable members are putatively linked to adaptive differences and phenotypic variation among accessions, further functional experiments are required to establish definitive causal relationships.

The Ka/Ks analysis also suggests that the *BnSWEET* family has not been subjected to completely uniform selective constraints during evolution. Most members had Ka/Ks values below 1, indicating that purifying selection remains the dominant force, which is consistent with the basic roles of SWEET proteins in sugar transport and plant growth and development. At the same time, however, some *BnSWEET* genes repeatedly showed Ka/Ks values greater than 1 in multiple accessions, suggesting that these members may have undergone stronger divergence. Similar findings are common in *SWEET* family studies from other species: duplicated genes often retain a relatively stable structural basis, while differences gradually emerge in expression pattern and function. In barley and cucumber, sequence evolution and expression divergence among clades have likewise been linked to functional differentiation after family expansion (Li et al., 2017; Yue et al., 2023). Therefore, the *BnSWEET* family in rapeseed appears to have remained largely conserved overall, while some members have undergone further adaptive divergence.

The high level of collinearity among rapeseed accessions indicates that the genomic regions containing *BnSWEET* genes are generally stable, although local variation is still retained at some loci. This pattern of overall conservation with local divergence is not uncommon in polyploid crops. Compared with other members, *BnSWEET5* deserves particular attention. Different haplotypes of this gene were significantly associated with seed oil content, silique length, and germination rate, and they also differed in expression level and gene structure. Our identification of 28 sequence variants (27 SNPs and one InDel) within *BnSWEET5* provides a molecular-level explanation for the observed trait differences among cultivars. Although the gene structure remained highly conserved among the eight core genomes used for evolutionary analysis, a large-scale analysis of 1,626 rapeseed accessions revealed significant phenotypic divergence among haplotypes. Specifically, variants in *hap3* (such as the InDel or key SNPs) may alter protein secondary structure or stability, potentially impairing sugar transport activity. This offers a plausible explanation for the lower oil content and reduced germination vigor observed in accessions carrying this haplotype. Furthermore, expression analysis showed that *hap2* levels were significantly higher than those of *hap1*, suggesting that variations in the promoter or UTR regions might modulate cis-element binding efficiency, thereby regulating energy partitioning during seed development at the transcriptional level. In other words, the natural variation of *BnSWEET5* is reflected not only at the sequence level but potentially extends to its transcriptional regulation. Given the established role of SWEET proteins in sugar transport and carbon allocation, *BnSWEET5* is hypothesized to play a role in modulating multiple agronomic traits by potentially affecting assimilate partitioning during reproductive development and early seed germination. Similar patterns have been observed in several fruit and seed crops, where variation in *SWEET* genes is often correlated with developmental processes, source–sink transport, or trait-related expression differences (Xie et al., 2019; Chen et al., 2024). This suggests that certain individual *SWEET* loci might be linked to agronomic performance. However, it should be noted that these associations remain speculative based on our current bioinformatic and expression data. Further functional validation, such as CRISPR/Cas9-mediated knockout or heterologous expression, is required to definitively establish the causal relationship between *BnSWEET5* variation and specific phenotypic traits.

Transcriptome analysis further showed clear expression divergence of *BnSWEET* genes across tissues, developmental stages, and abiotic stress conditions. Some genes in Group I and Group III were highly expressed in leaves and roots and responded strongly to salt, drought, and osmotic stress. In contrast, most members in Group II and Group IV showed generally low expression and were induced only under specific conditions. These expression differences suggest that *BnSWEET* genes from different clades have likely undergone different degrees of functional division. Similar patterns have been reported in cucumber, barley, *Brassica juncea*, sweet sorghum, watermelon, and *Hemerocallis citrina*, where different *SWEET* clades or members often show tissue-preferential expression and distinct responses to salt, drought, cold, or heat stress (Miao et al., 2017; Cao et al., 2024; Heng et al., 2024; Pan et al., 2024; Zhang et al., 2024a). In addition, previous studies have shown that prolonged heat stress during flowering in *B. napus* also causes marked transcriptional changes in multiple *SWEET* genes (Kourani et al., 2025). Taken together with our results, expression divergence is likely one of the important reasons why duplicated SWEET members have been retained and gradually specialized in function. The RT-qPCR results demonstrate that the *BnSWEET* family members possess highly heterogeneous temporal expression patterns under salt, alkali, and drought stresses. This may reflect the diversity of the family in terms of functional differentiation, stress signal perception, and evolutionary adaptation strategies. Similar expression heterogeneity has been reported in studies on the SWEET family response to cold stress in *Prunus mume* (Wen et al., 2022) and Rosa (Song et al., 2023), suggesting that this may be a conserved regulatory mechanism employed by plant SWEET families to cope with abiotic stress.

In summary, our pan-genome analysis revealed that the *BnSWEET* gene family in *Brassica napus* is largely conserved at the structural level, while certain members exhibit notable variation in presence/absence, sequence, and expression. Evolutionary analyses indicated that most genes are under purifying selection, whereas a subset has undergone positive selection, suggesting adaptive divergence. BnSWEET5, in particular, shows natural variation in sequence, structure, and expression that correlates with key agronomic traits, highlighting its potential functional significance. Transcriptome and RT-qPCR analyses further demonstrated tissue-specific and stress-responsive expression patterns, supporting functional differentiation among family members. Overall, these findings suggest that while the *BnSWEET* family maintains core sugar transport functions, variable and stress-responsive members contribute to phenotypic diversity and environmental adaptation in rapeseed, providing valuable insights for future functional studies and crop improvement.

## 4 Materials and methods

### 4.1 Identification of the SWEET gene family in B. napus

In this study, eight *B. napus* accessions representing three ecotypes, namely ZS11, Gangan, Zheyou7, Shengli, Tapidor, Quinta, Westar, and No2127, were used for bioinformatic analysis. Genome sequences of these accessions were obtained from Song et al. [31]. To identify SWEET family members, the Hidden Markov Model (HMM) profile of the MtN3/saliva domain (PF03083), which is the characteristic conserved domain of SWEET proteins, was downloaded from the Pfam database (http://pfam.xfam.org/) (Finn et al., 2014). HMMER 3.3.2 was then employed to search candidate SWEET proteins in the eight *B. napus* genomes using an E-value threshold of 10E−5 (Potter et al., 2018). The obtained candidate sequences were further submitted to the SMART database (http://smart.embl-heidelberg.de/) [48] to confirm the presence of the conserved MtN3/saliva domain. Genes that contained the characteristic SWEET domain and exhibited collinearity with *SWEET* genes among the eight *B. napus* genomes were finally identified as members of the *SWEET* gene family.

### 4.2 Phylogenetic analysis and presence/absence variation of the BnSWEET gene family

Phylogenetic analysis was performed using SWEET protein sequences from *Arabidopsis thaliana* and *B. napus*. Multiple sequence alignment was conducted with MUSCLE, and a phylogenetic tree was constructed using MEGA11 (Tamura et al., 2021). The resulting tree was visualized with Evolview v3 (Subramanian et al., 2019). To evaluate the genomic distribution of the BnSWEET family, the identified genes were classified into five categories based on their presence/absence variation (PAV) across the eight *B. napus* accessions: (1) core genes, present in all 8 accessions; (2) near-core genes, present in 6 or 7 accessions (missing in 1–2 accessions); (3) dispensable genes, present in 2 to 5 accessions; (4) private genes, present in only 1 accession; and (5) unannotated genes, referring to the 37 SWEET members identified in the individual genomes of the eight accessions that lacked corresponding gene models or annotations in the final integrated pan-genome assembly. Presence/absence variation (PAV) data for *SWEET* genes were obtained from Song et al (Song et al., 2020). A heatmap showing the distribution of *SWEET* genes across the eight *B. napus* accessions was generated in R v4.0.3 using the ComplexHeatmap package (Gu et al., 2016).

### 4.3 Calculation of Ka/Ks values

Protein sequences and coding sequences (CDSs) of *BnSWEET* genes from the eight *B. napus* genomes were retrieved from Song et al (Song et al., 2020). After sequence alignment, nonsynonymous substitution rates (Ka), synonymous substitution rates (Ks), and Ka/Ks ratios were calculated using KaKs Calculator (Zhang, 2022). To ensure statistical reliability, gene pairs with Ks < 0.001 were excluded to avoid artificial inflation caused by near-zero denominators, and those with Ks > 1.5 were removed to avoid substitution saturation. The complete raw data and filtering status are provided in **Supplementary Table 4**. Ridgeline plots showing the distribution of Ka/Ks values were produced using the ggridges and ggplot2 packages in R v4.0.3 (Wickham, 2011). In addition, a heatmap was generated with ComplexHeatmap (Gu et al., 2016) to illustrate the proportion of *BnSWEET* genes with Ka/Ks values greater than 1.

### 4.4 Collinearity analysis of SWEET family genes

This analysis used data from eight *B. napus* accessions, namely Gangan, No2127, Quinta, Shengli, Tapidor, Westar, Zheyou7, and ZS11. The genomic data were obtained from the BnPIR database (https://doi.org/10.1111/pbi.13491) (Song et al., 2021), including the whole-genome sequences and GFF3 annotation files for each accession. In TBtools, the Advanced Circos tool was used to perform intraspecific synteny analysis of the *BnSWEET* genes in *B. napus*, while the MCScanX module was employed to analyze the synteny relationships among Gangan, No2127, Quinta, Shengli, Tapidor, Westar, Zheyou7, and ZS11 (Wang et al., 2012).

### 4.5 Identification of SWEET gene duplication modes

Based on the results of whole-genome protein sequence self-alignment (Self-BlastP) (Buchfink et al., 2015), the identified *BnSWEET* genes were precisely classified into five duplication types using the MCScanX toolkit (Wang et al., 2012) and its accompanying duplicate_gene_classifier algorithm: whole-genome duplication (WGD)/segmental, tandem, proximal, dispersed, and singleton.

### 4.6 Haplotype analysis and phenotype data

The SNP and Indel data and phenotypic data (such as Flowering time, thousand seed weight (TSW) and Seed oil content(SOC) for different rapeseed germplasm were downloaded from the multi-focus model variation toolbar on the BnVIR online website (https://yanglab.hzau.edu.cn/BnVIR) (Yang et al., 2022).The LD heatmap was generated and visualized using the integrated LD toolkit within the BnVIR platform to define the LD block structure and evaluate potential linkage interference from neighboring genomic regions.

### 4.7 Structural characterization analysis of SWEET genes in B. napus

The genome annotation file in GFF format was downloaded from https://doi.org/10.1111/pbi.13491 (accessed on 5 July 2025). Conserved motifs in BnSWEET proteins were identified using MEME (Bailey et al., 2015). Genes encoding proteins with SNP variations that significantly affected expression were further analyzed. TBtools II v2.301 was then used to integrate the results from the motif analysis, SNP-associated expression analysis, and the GFF annotation file to generate gene structure diagrams (Chen et al., 2020).

### 4.8 Transcriptome expression analysis of BnSWEET genes under different conditions

To investigate the expression patterns of *BnSWEET* genes under different conditions, transcriptome datasets were collected from published databases and previous studies. For abiotic stress-related transcriptome analysis, BnSWEET sequences were submitted to the BnIR database (Liu et al., 2021), and TPM values of *BnSWEET* genes in leaves and roots were retrieved under control (CK), salt stress (200 mmol·L^−1^ NaCl), drought (natural air-drying), freezing stress (−4 °C followed by recovery at 25 °C), cold stress (4 °C followed by recovery at 25 °C), heat stress (38 °C followed by recovery at 25 °C), and osmotic stress (300 mmol·L^−1^ mannitol). For developmental transcriptome analysis, gene expression data from five sowing stages in eight *B. napus* accessions were obtained from Song et al (Song et al., 2020), including T0 (24 days after sowing), T1 (54 days), T2 (82 days), T3 (115 days), and T4 (147 days), using ZS11 as the reference genome. The abiotic stress response profiles retrieved from the BnIR database were derived from three independent biological replicates, while the developmental expression dynamics were characterized using two biological replicates across multiple accessions and growth stages. TPM and FPKM values were normalized using log_10_(TPM + 1) or log_10_(FPKM + 1), respectively, and the resulting expression matrices were visualized with TBtools (Chen et al., 2020).

### 4.9 Weighted gene co-expression network analysis

To investigate gene co-expression patterns and potential regulatory networks across the five developmental stages (T0–T4), Weighted Gene Co-expression Network Analysis was performed using the R package “WGCNA” (Langfelder and Horvath, 2008). After filtering low-expression genes, an optimal soft-thresholding power of β=26 was selected to construct the adjacency matrix, thereby ensuring a scale-free network topology. The adjacency matrix was subsequently transformed into a Topological Overlap Matrix (TOM) to minimize noise and false-positive correlations. Hierarchical clustering was conducted based on the TOM dissimilarity measure, and co-expression modules were identified and merged employing the dynamic tree cut algorithm. To discern stage-specific functional modules, module-trait relationships were evaluated by calculating Pearson correlation coefficients between module eigengenes (MEs) and the respective developmental stages. Finally, the top 30 hub genes based on high intramodular connectivity, together with the target gene BnSWEET47, were extracted from the highly correlated palevioletred3 module, and the core regulatory network was visualized using Cytoscape software (v2.8) (Smoot et al., 2011).

### 4.10 Expression analysis of BnSWEET genes under different abiotic stresses

The *B. napus* cultivar ZS11 was used for stress-induced expression analysis. Seeds were provided by the research group of Professor Kede Liu, Huazhong Agricultural University, China. Seeds were germinated in distilled water for 4 days, and uniformly growing seedlings were transferred to Hoagland nutrient solution for 14 days. The seedlings were then subjected to 1.0% (w/v) NaCl, 0.7% (w/v) NaHCO_3_, and 20% (w/v) PEG 6000 prepared in Hoagland solution. Samples for salt and drought stress analyses were collected at 0, 6, 12, and 24 h for RNA extraction and subsequent expression analysis.

Primers were designed using qPrimerDB (https://biodb.swu.edu.cn/qprimerdb/) (Li et al., 2024), and the reference gene used in this study is listed in **Table 1**. Total RNA was extracted using an RNA extraction kit, and first-strand cDNA was synthesized using HiScript II Q RT SuperMix for qPCR. Quantitative PCR was performed with ChamQ Universal SYBR qPCR Master Mix. Each 20 μL reaction mixture contained 1 μL cDNA, 0.4 μL of each forward and reverse primer, 10 μL SYBR mix, and sterile ddH_2_O to a final volume of 20 μL. The PCR program consisted of an initial denaturation at 95 °C for 30 s, followed by 40 cycles of 95 °C for 10 s and 60 °C for 22 s. Relative expression levels were calculated using the 2^−ΔΔCt^ method, and the results were visualized with Prism 10. Based on preliminary experiments under four abiotic stress treatments, ACT7 was selected as the internal reference gene (Chen et al., 2023; Xue et al., 2025).To ensure the reliability of our findings, RT-qPCR validation was performed for each gene with three independent biological replicates and three technical replicates.

**Table 1:** qPCR primer sequence of *BnSWEET* genes.

Gene name	Forward primer (5’→3’)	Reverse primer (3’→5’)
*BnSWEET4*	TGGAGCCACTTTCCAGCT	AACCAACGCAACCACAGC
*BnSWEET5*	GGTGACGGAACCGGTGAT	GGCTTTGTGCGGCTTCAG
*BnSWEET26*	ATCTTCGCCGCCGTCTTG	CACAGTGGCTGCGATACCA
*BnSWEET32*	CCCTATGTGTCAGCGCTCT	AAGCATCCCACGGCGTTT
*BnSWEET37*	TTGTTCCGGTGGTGGTCA	AGGCCGAAACTTCCGACG
*BnSWEET51*	ATTGCGGCCACGAGAAGT	CGCTCTTTGTCGCCACCA
*ACT7*	GCTGACCGTATGAGCAAAG	AAGATGGATGGACCCGAC

## 4 Conclusion

In conclusion, this study provides a comprehensive pan-genome analysis of the *SWEET* gene family in *Brassica napus*, identifying 96 *BnSWEET* genes and revealing significant Presence/Absence Variations (PAVs) and evolutionary divergence. Notably, this work prioritizes specific candidate genes for future genetic improvement: *BnSWEET5* is identified as a primary target for quality breeding due to the strong association of its elite haplotypes with high oil content and germination vigor; *BnSWEET47* emerges as a core regulatory hub for coordinating sugar partitioning and secondary metabolism; and *BnSWEET26* (PAV-specific) and *BnSWEET37* (positively selected) represent valuable genetic resources for environmental adaptation. These findings provide a solid basis for the precision screening of rapeseed germplasm using identified elite haplotypes and key PAV variants, thereby offering a practical genetic improvement pathway for developing high-yielding, high-quality, and stress-resilient varieties.

## Data Availability

The original contributions presented in the study are included in the article/**Supplementary Material**. Further inquiries can be directed to the corresponding author.
