# Supplementary material for: Pan-genome analysis and abiotic stress expression of the SWEET gene family in Brassica napus
Source: Front Plant Sci. 2026 May 26;17:1846550. doi: 10.3389/fpls.2026.1846550 (PMC13246404; doi:10.3389/fpls.2026.1846550)
Supplement: Supplementary file 1 [file DataSheet1.zip › Figure S1.pdf]

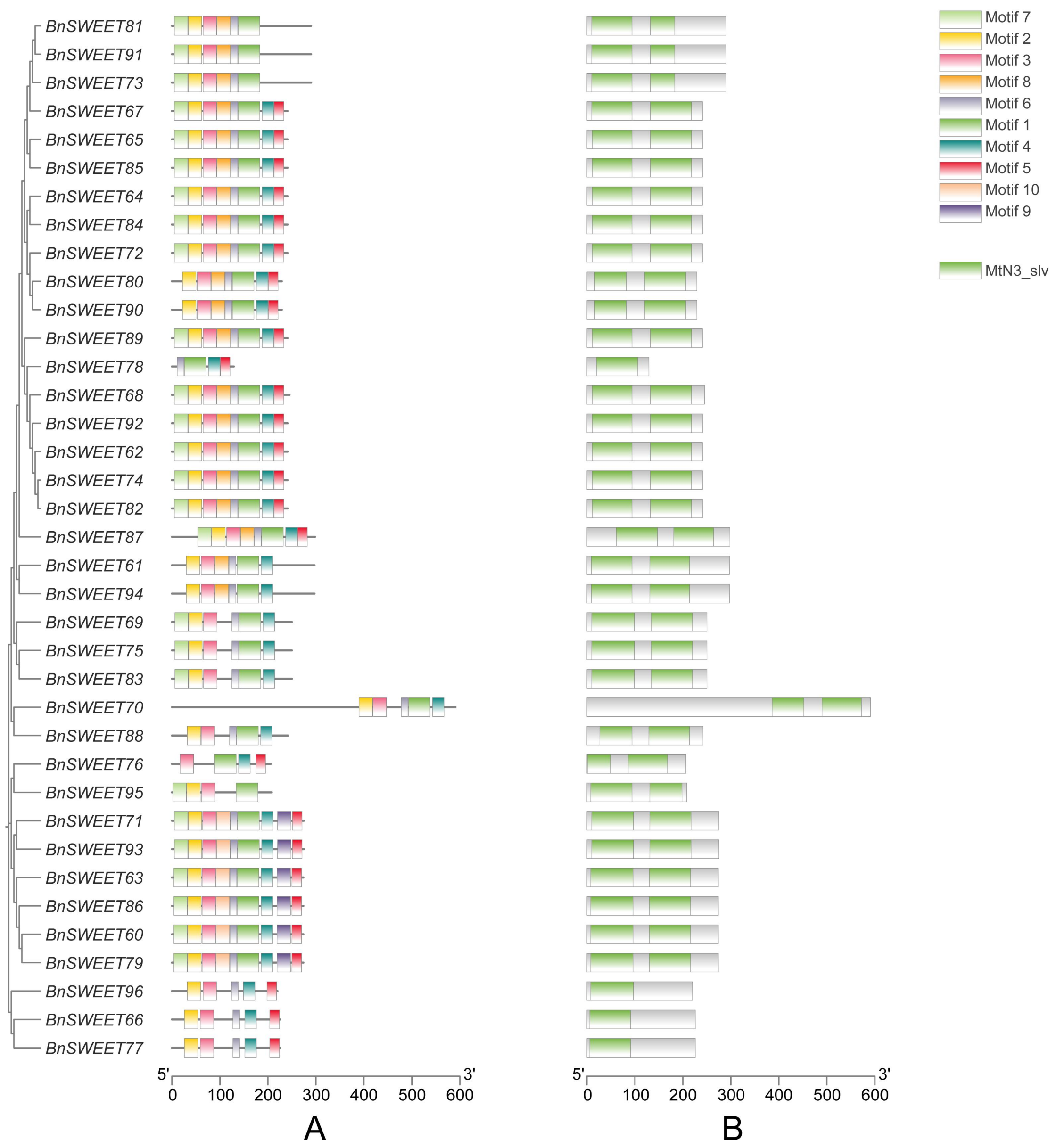

Figure S1. Phylogenetic relationships, conserved motifs, and domain architectures of 37 newly identified *SWEET* genes (*BnSWEET60–96*) in the *Brassica napus* pan-genome.(A) Gene structure. By using MEME Suite to search for motifs in the *BnSWEET* gene, nine motifs were found to be conserved across different *B. napus* varieties. (B) Conserved domains: Demonstrates the stability of the MtN3 (MtN3/saliva) domain, which is a core characteristic of the *SWEET* family.
