# Supplementary figures and images for: Pan-genome analysis and abiotic stress expression of the SWEET gene family in Brassica napus
Source: Front Plant Sci. 2026 May 26;17:1846550. doi: 10.3389/fpls.2026.1846550 (PMC13246404; doi:10.3389/fpls.2026.1846550)

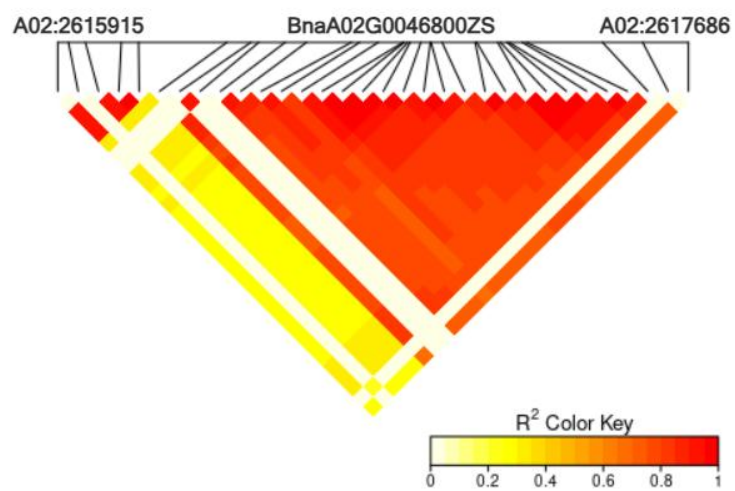

Figure S2. Linkage disequilibrium (LD) heatmap of the *BnSWEET5* locus on chromosome A02.

Supplement: Supplementary file 1 [file DataSheet1.zip › Figure S2.pdf]
